# Supplementary material for: Gram-negative ESKAPE bacteria bloodstream infections in patients during the COVID-19 pandemic
Source: PeerJ. 2023 Mar 29;11:e15007. doi: 10.7717/peerj.15007 (PMC10066687; doi:10.7717/peerj.15007)
Supplement: Supplemental Information 2 [file peerj-11-15007-s002.pdf]

Uncropped Gels
Acinetobacter baumannii

Acinetobacter baumannii (*n* = 39, pulsotypes = 16)
Hospital General de México Dr. Eduardo Liceaga 2020

Molecular weight
PFGE lambda ladder

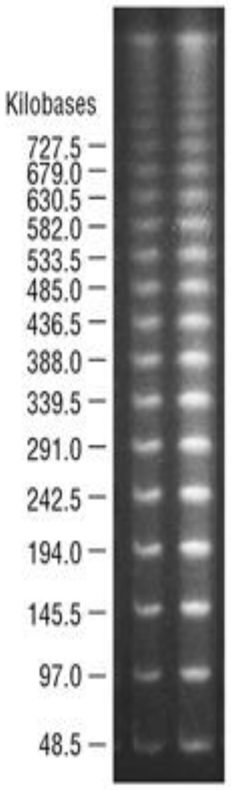

Gel 1 *Acinetobacter baumannii*

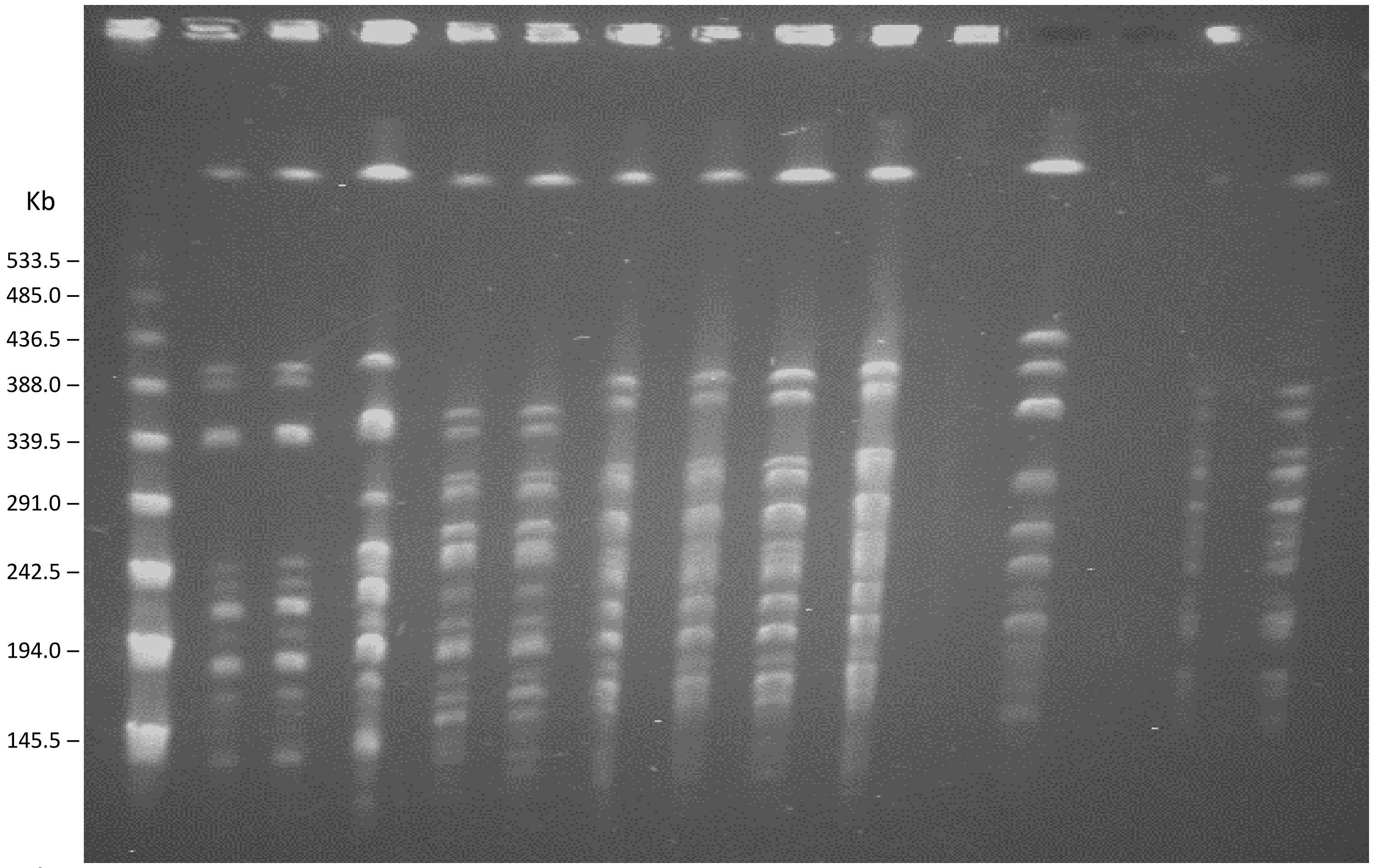

Gel 2 *Acinetobacter baumannii*

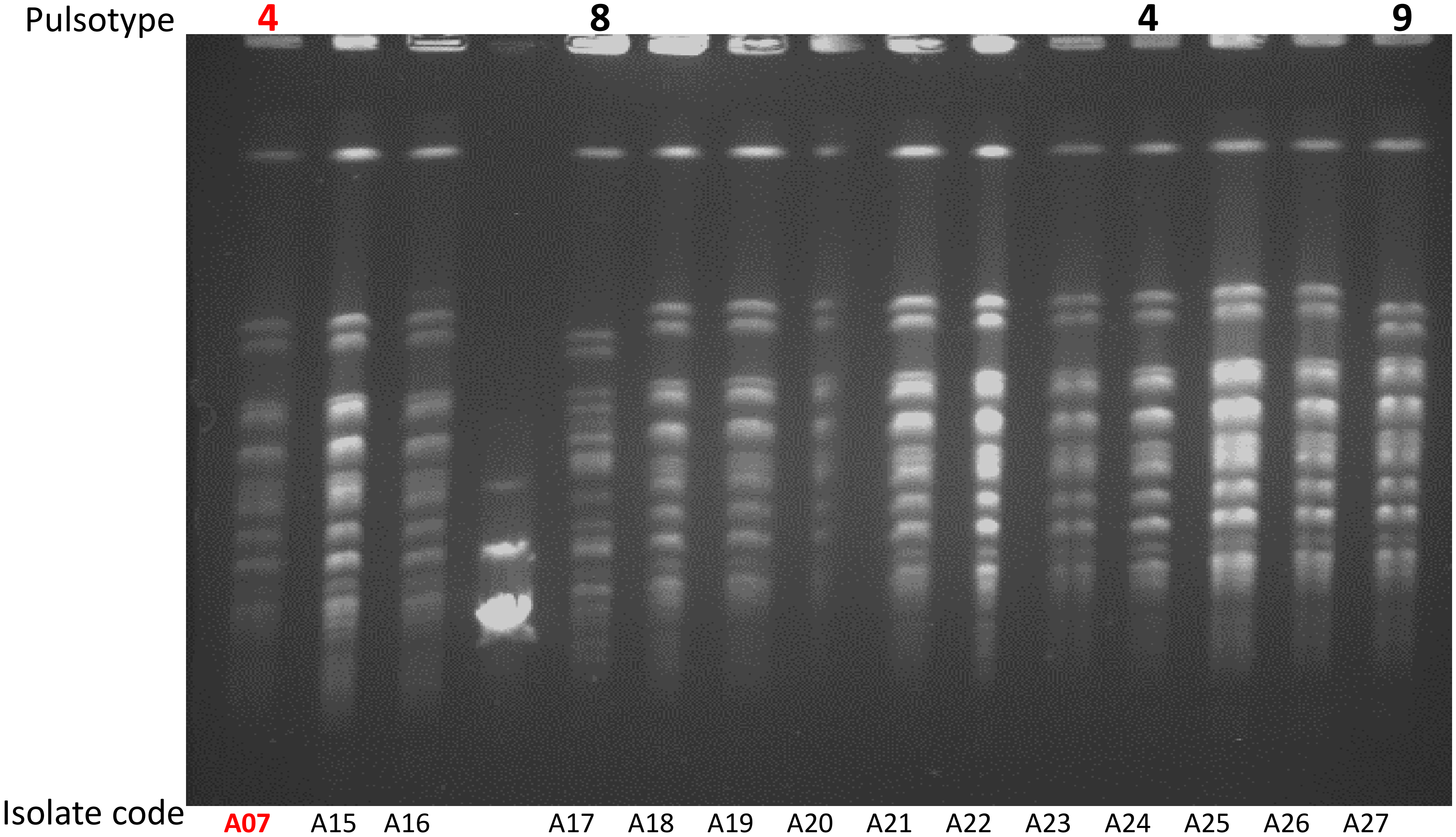

A07 = Outbreak strain- PFGE patthern (pulsotype 4)

Molecular weight PFGE lambda ladder

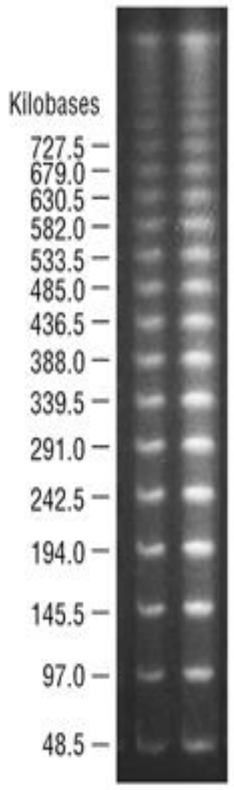

Gel 3 *Acinetobacter baumannii*

Pulsotype **MW** **10** **11** **12** **13** **14** **15** **16**

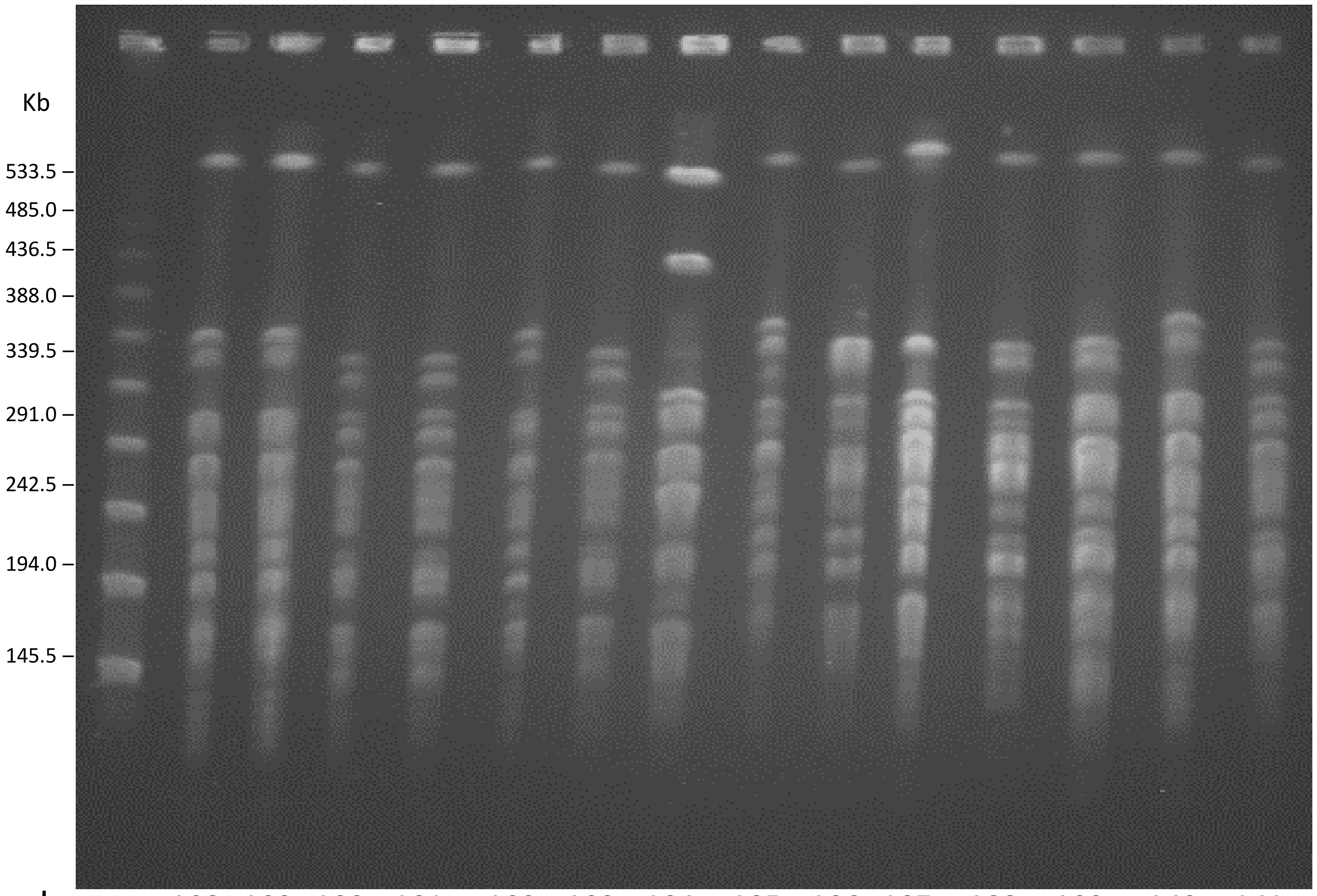

Isolate code A28 A29 A30 A31 A32 A33 A34 A35 A36 A37 A38 A39 A40 A41

Gel 4 *Acinetobacter baumannii*

Pulsotype **MW** **7**

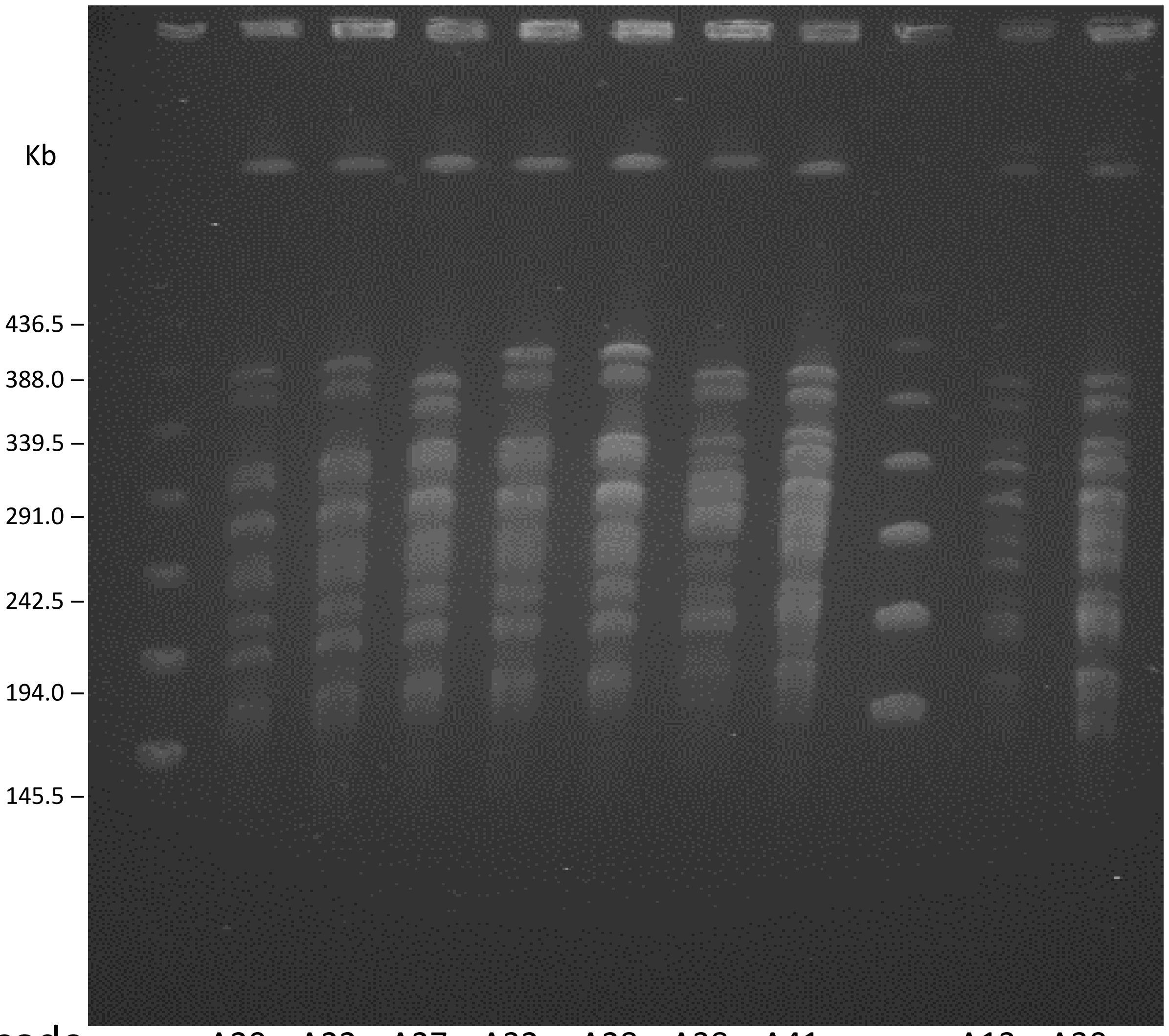

Isolate code A20 A22 A27 A32 A28 A38 A41 A13 A30

Gel 5 *Acinetobacter baumannii*

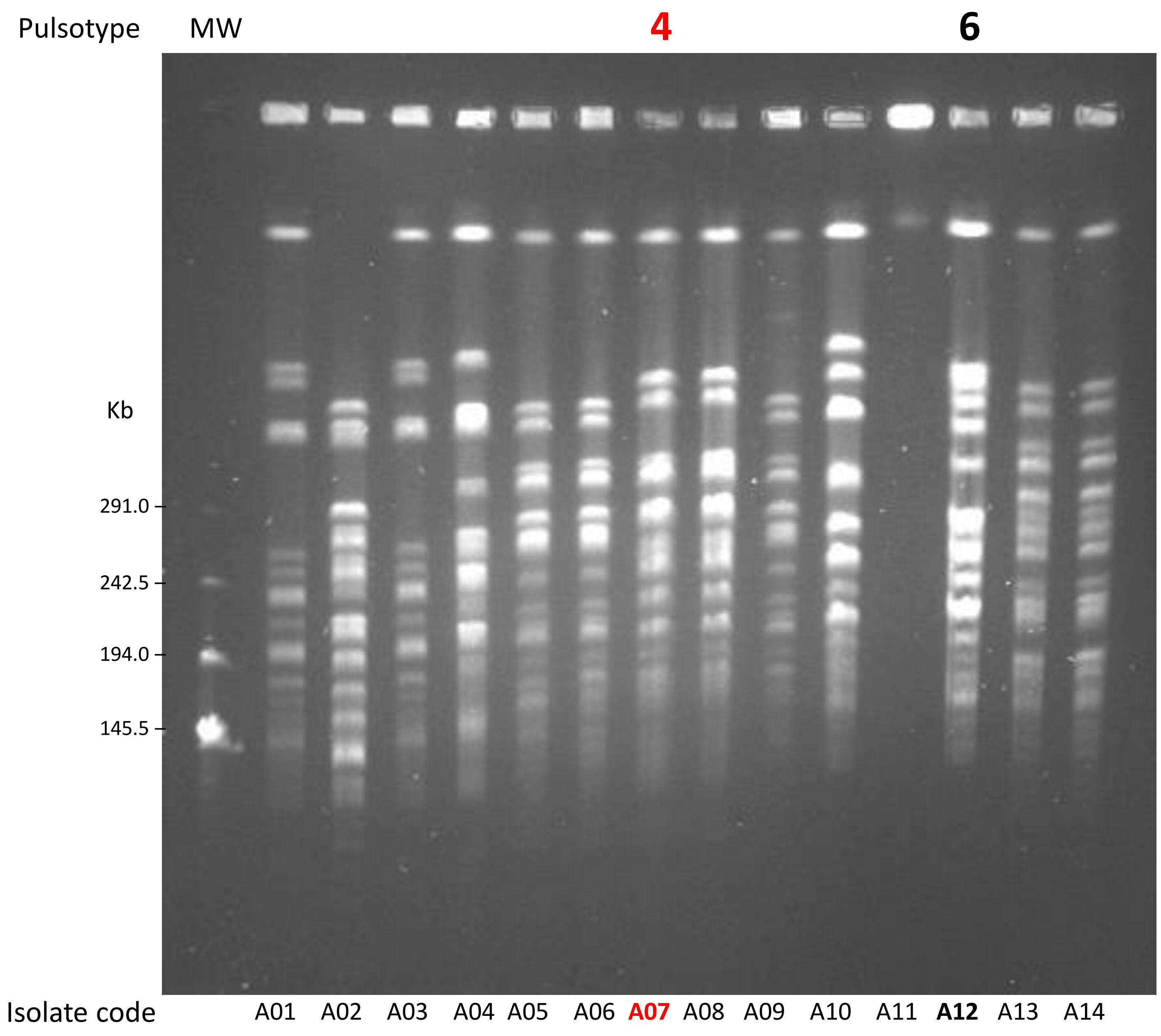

A07 = Outbreak strain- PFGE patthern (pulsotype 4)

Uncropped Gels
Klebsiella pneumoniae

Klebsiella pneumoniae (n = 29, pulsotypes = 25)
Hospital General de México Dr. Eduardo Liceaga 2020

Gel 1 *Klebsiella pneumoniae*

Pulsotype **WM 4 1 2 3 5 4 7 7**

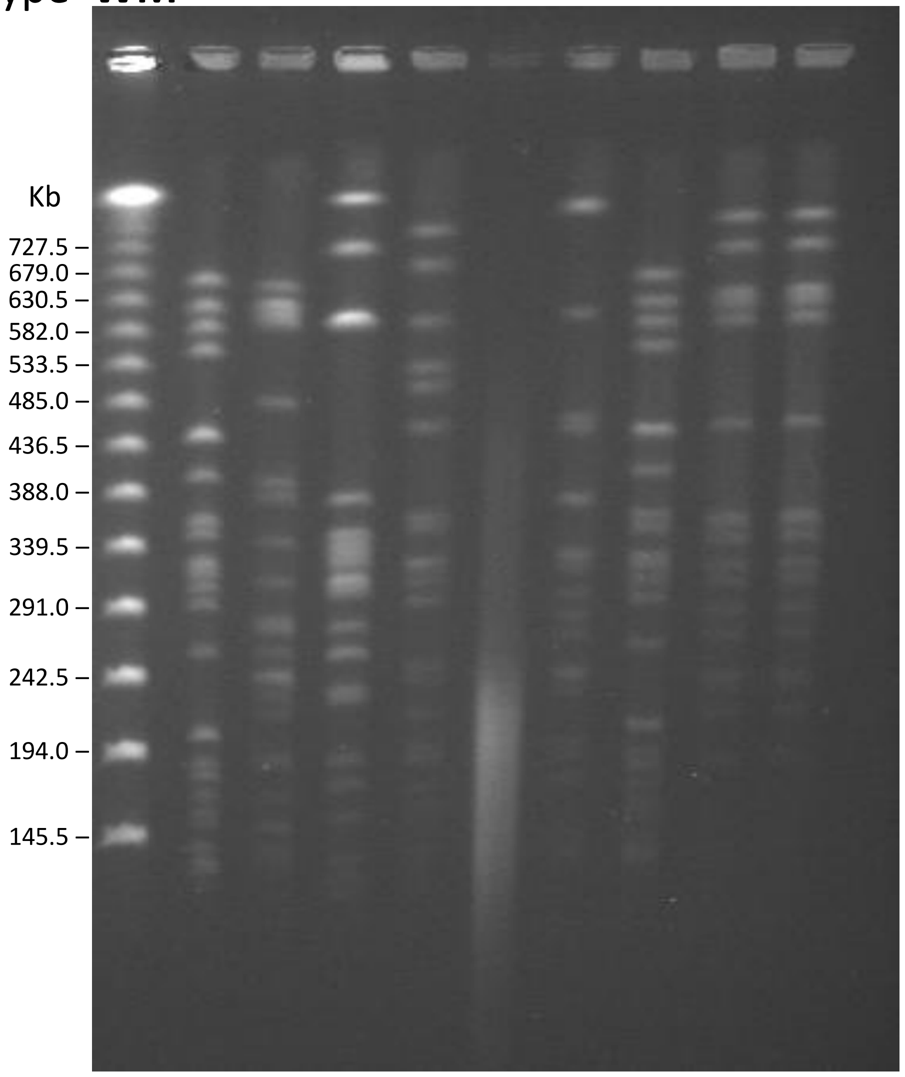

Isolate code K1 K2 K3 K4 K5 K6 K8 K9 K10

Molecular weight
PFGE lambda ladder

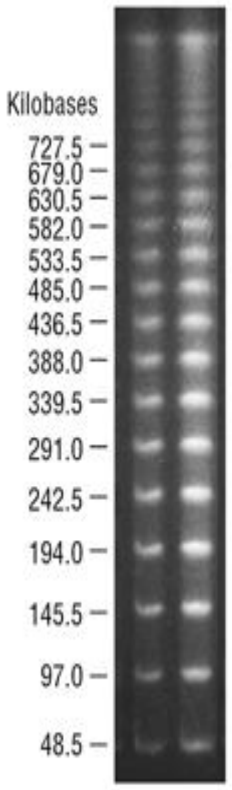

Gel 2 *Klebsiella pneumoniae*

Pulsotype **MW 9 8 6 10 11 15 12 13 14 16 17 18 17**

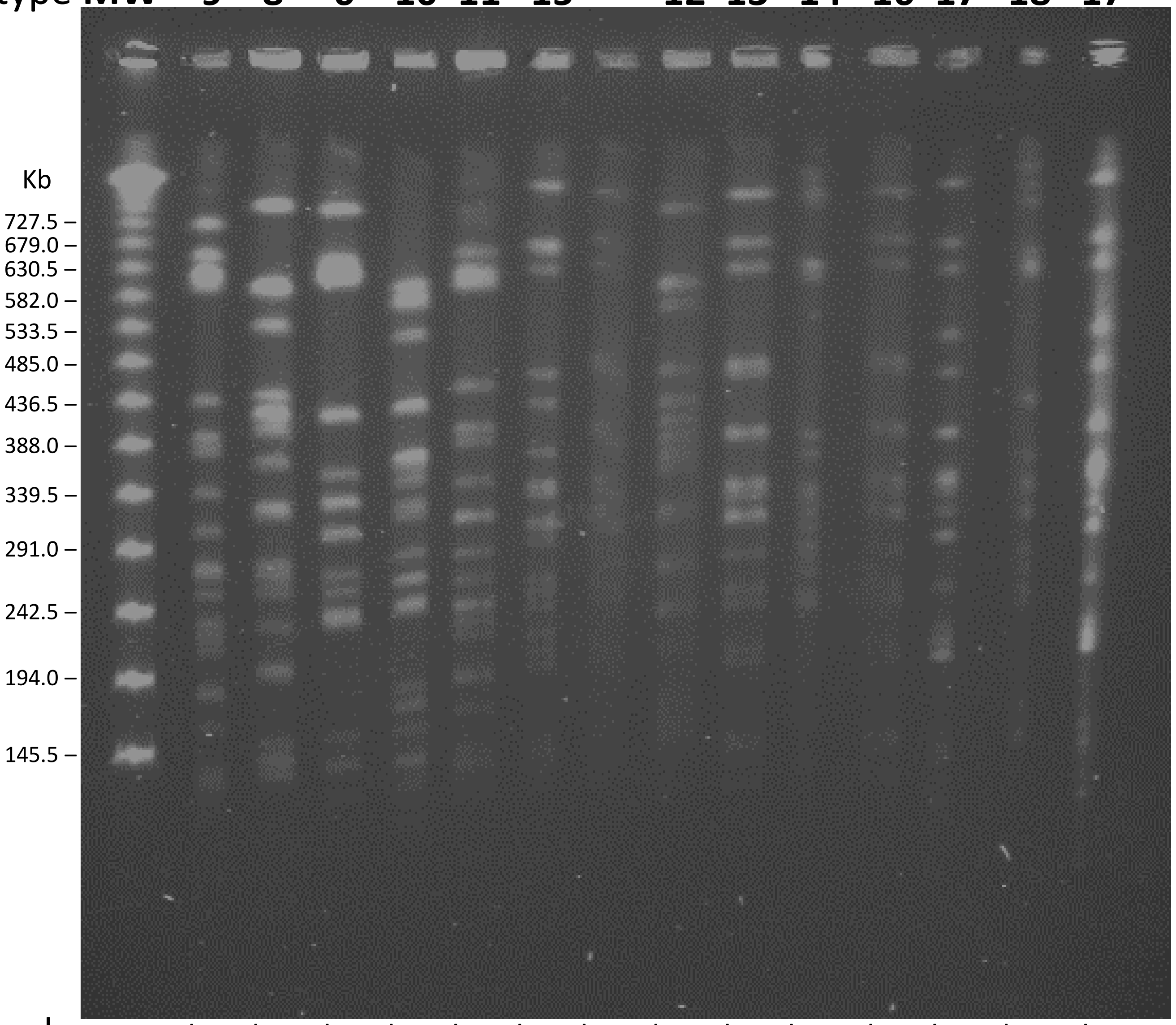

Isolate code k11 k12 k13 k14 k16 k17 k18 k19 k20 k21 k22 k24 k25 k26

Gel 3 *Klebsiella pneumoniae*

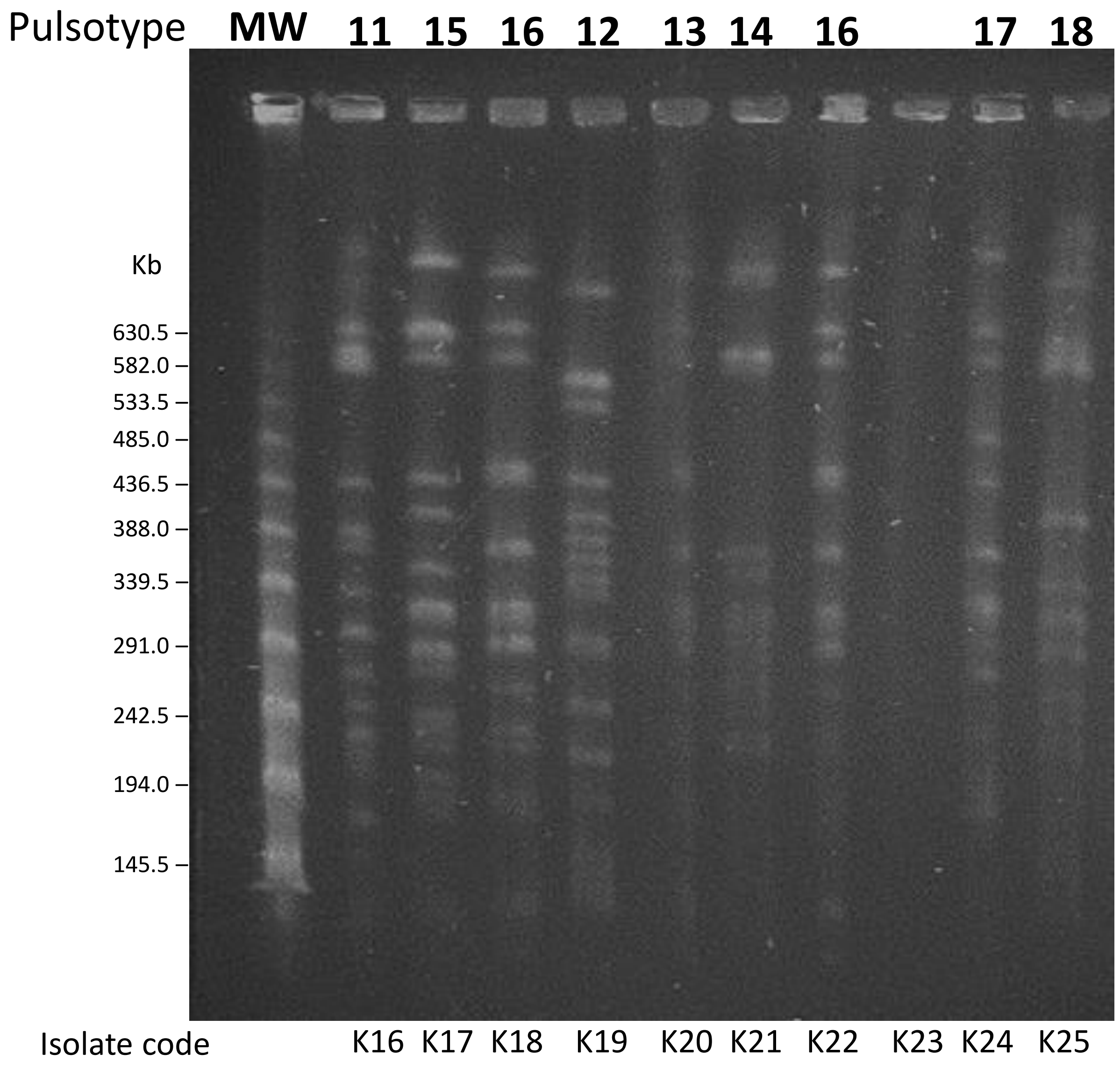

Gel 4 *Klebsiella pneumoniae*

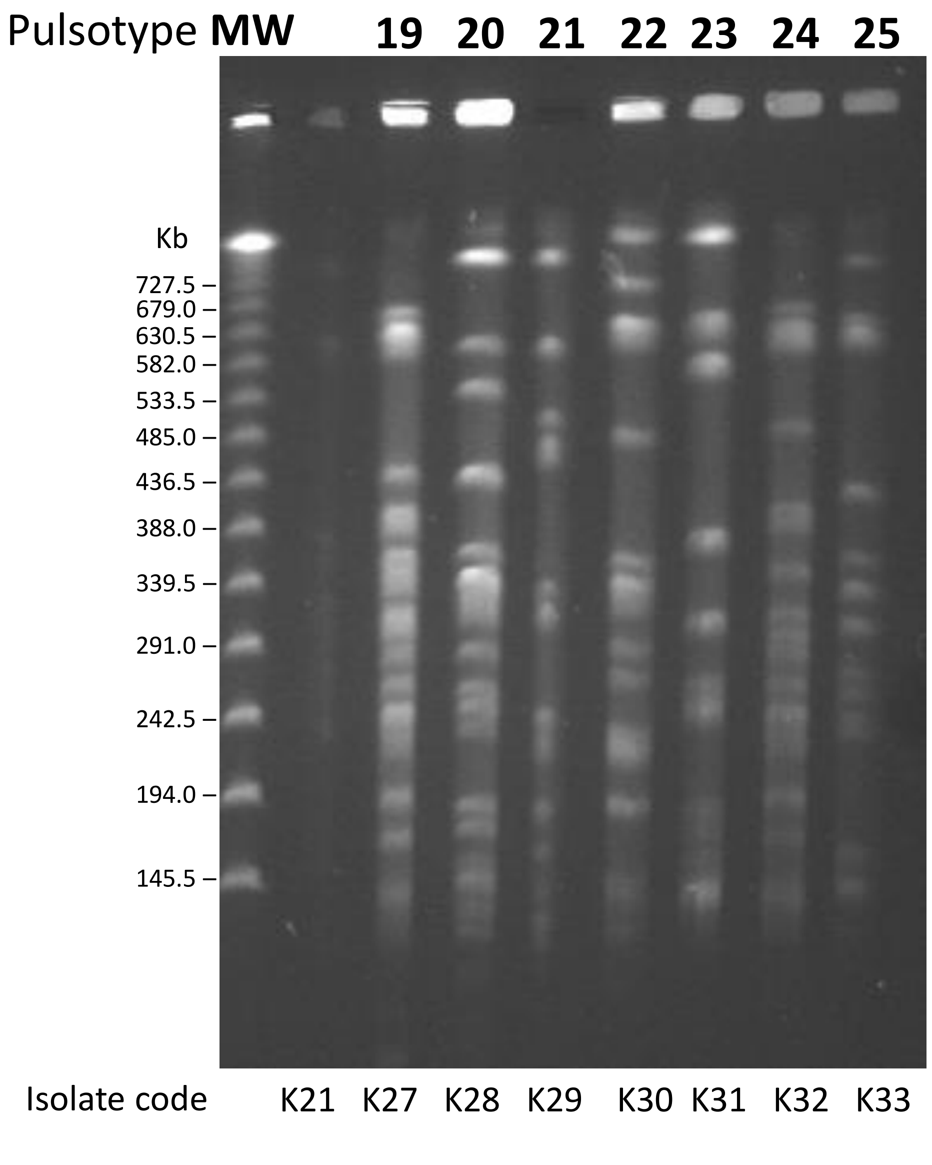

Uncropped Gels
Pseudomonas aeruginosa

Pseudomonas aeruginosa (n = 21, pulsotypes = 20)
Hospital General de México Dr. Eduardo Liceaga 2020

Molecular weight
PFGE lambda ladder

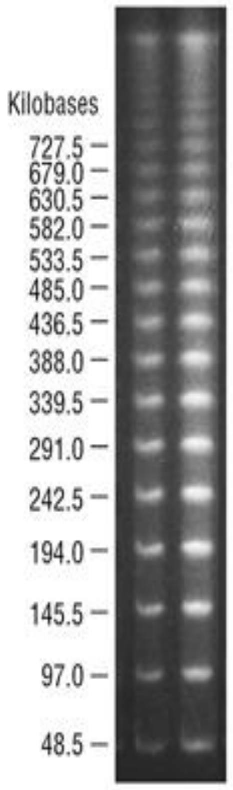

Gel 1 ***Pseudomonas aeruginosa***

Pulsotype **MW 3 1**

Kb
339.5 –
291.0 –
242.5 –
194.0 –
145.5 –
97.0 –
48.5 –

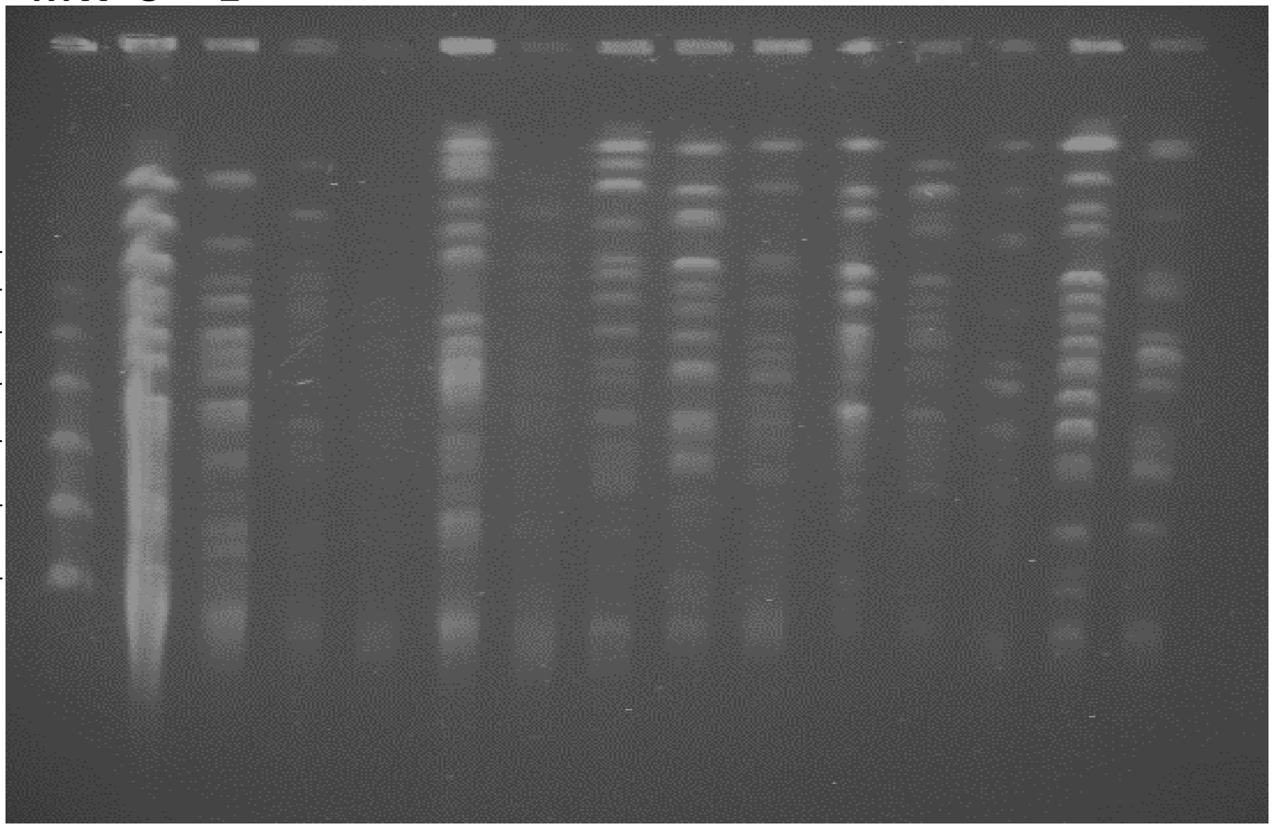

Isolate code **P01 P02** P03 P04 P05 P10 P11 P12 P13 P14 P15 P16 P17 P18

Gel 2 ***Pseudomonas aeruginosa***

Pulsotype **MW 2 4 5 6 7 8 9 10 11 12 13 14**

Kb
436.5 –
388.0 –
339.5 –
291.0 –
242.5 –
194.0 –
145.5 –
97.0 –
48.5 –

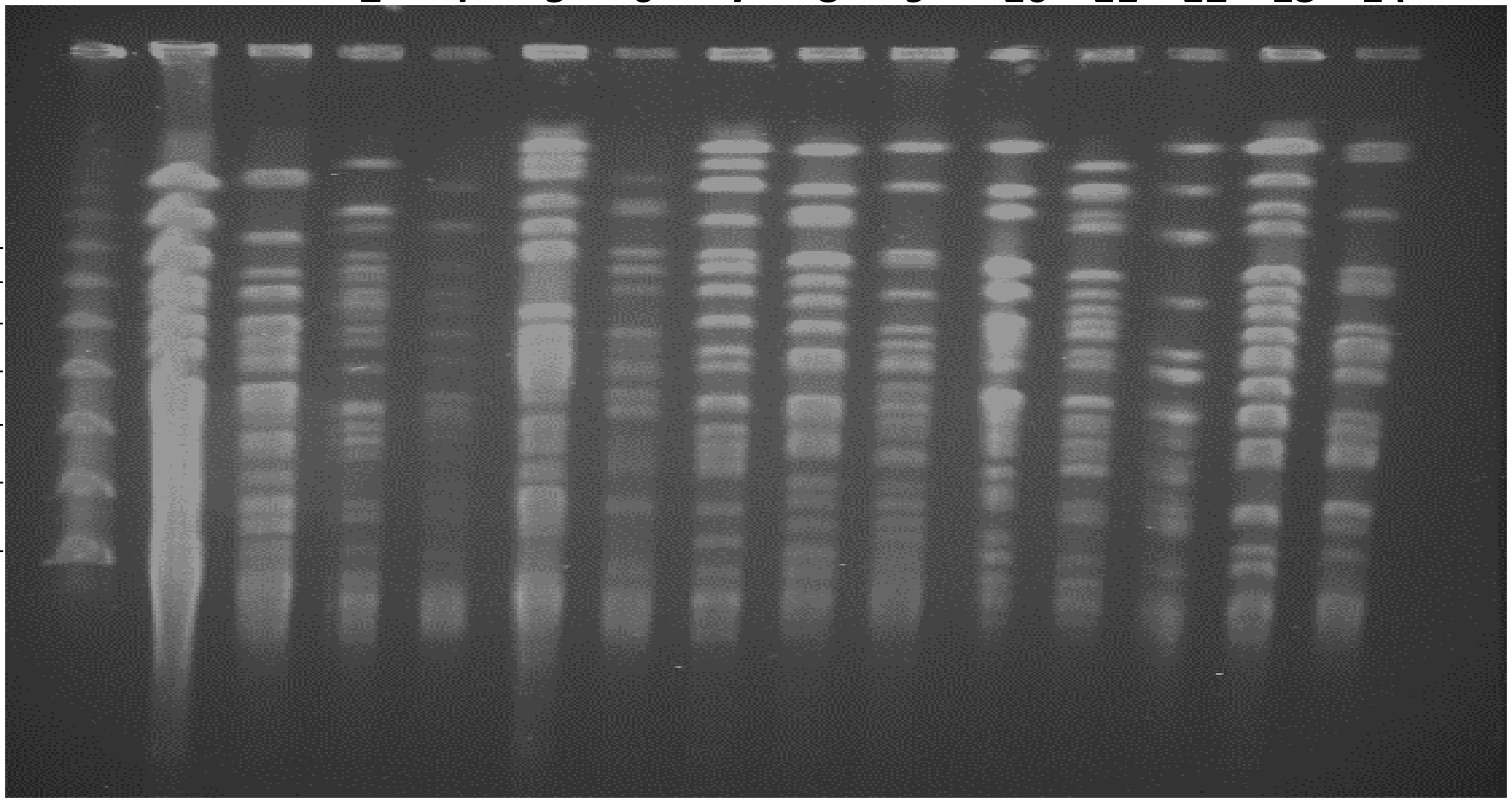

Isolate code P01 P02 P03 P04 P05 P10 P11 P12 P13 P14 P15 P16 P17 P18

Gel 3 *Pseudomonas aeruginosa*

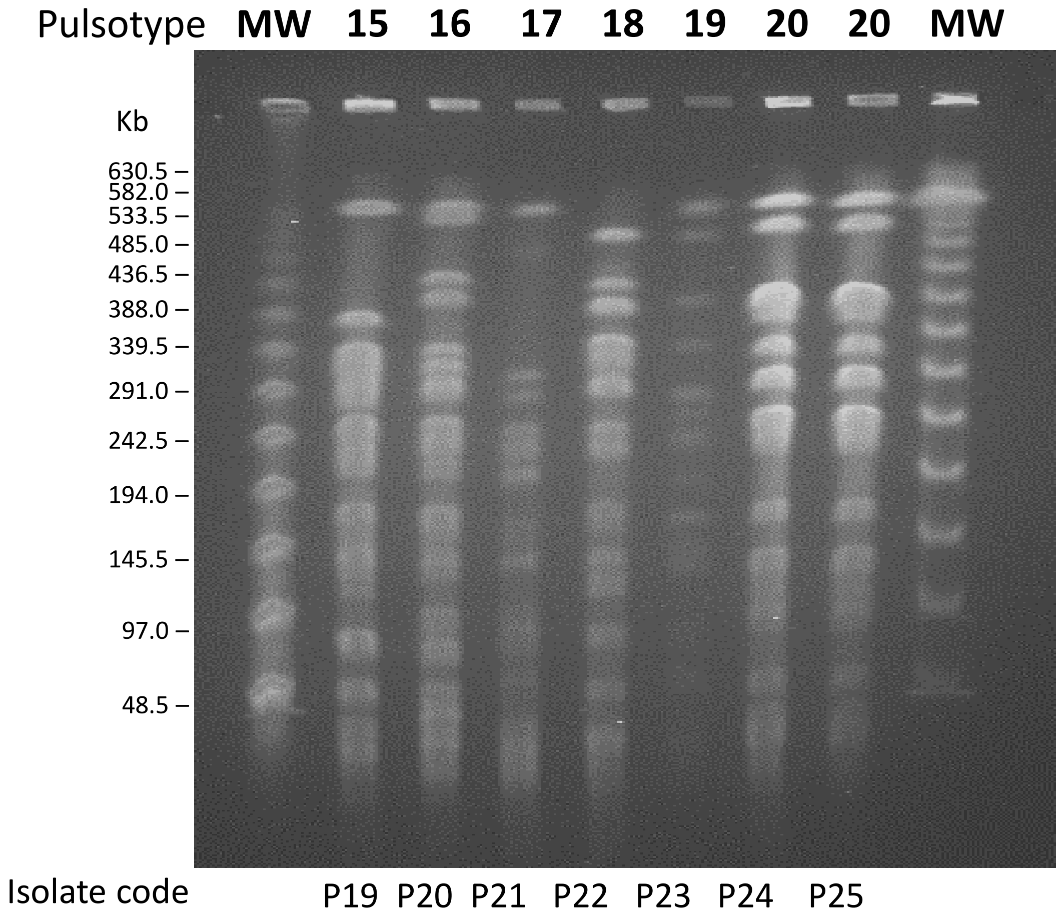

Uncropped Gels
Enterobacter spp

Enterobacter (n = 17, pulsotypes = 16)
Hospital General de México Dr. Eduardo Liceaga 2020

Gel 3 *Enterobacter*

Pulsotype **MW 1 3 6 6 7 MW**

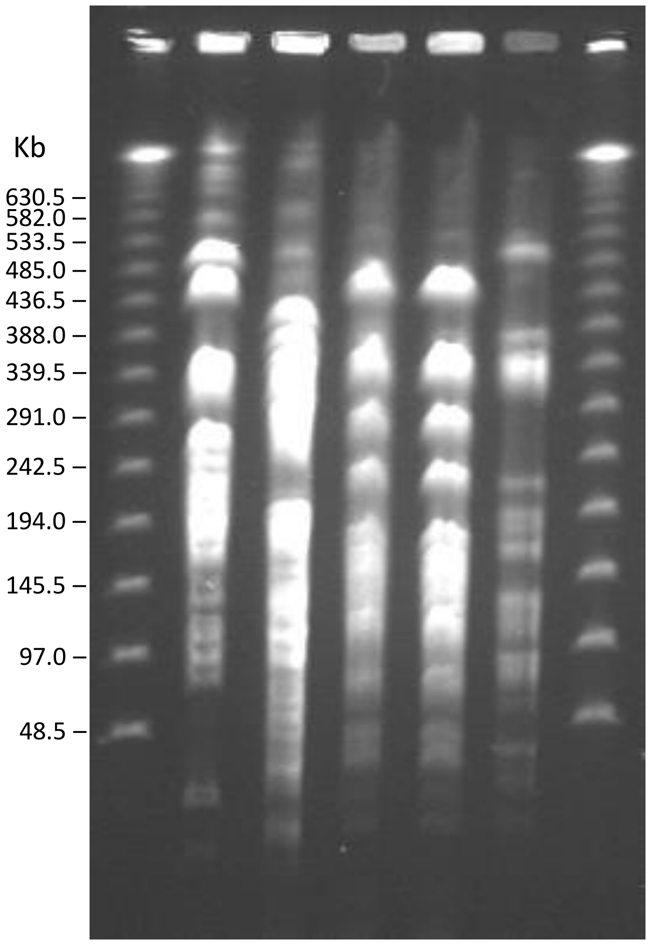

Isolate code E01 E03 E07 E08 E09

Gel 1 *Enterobacter*

Pulsotype **MW 8 9 10 11 12 13 14 15 16**

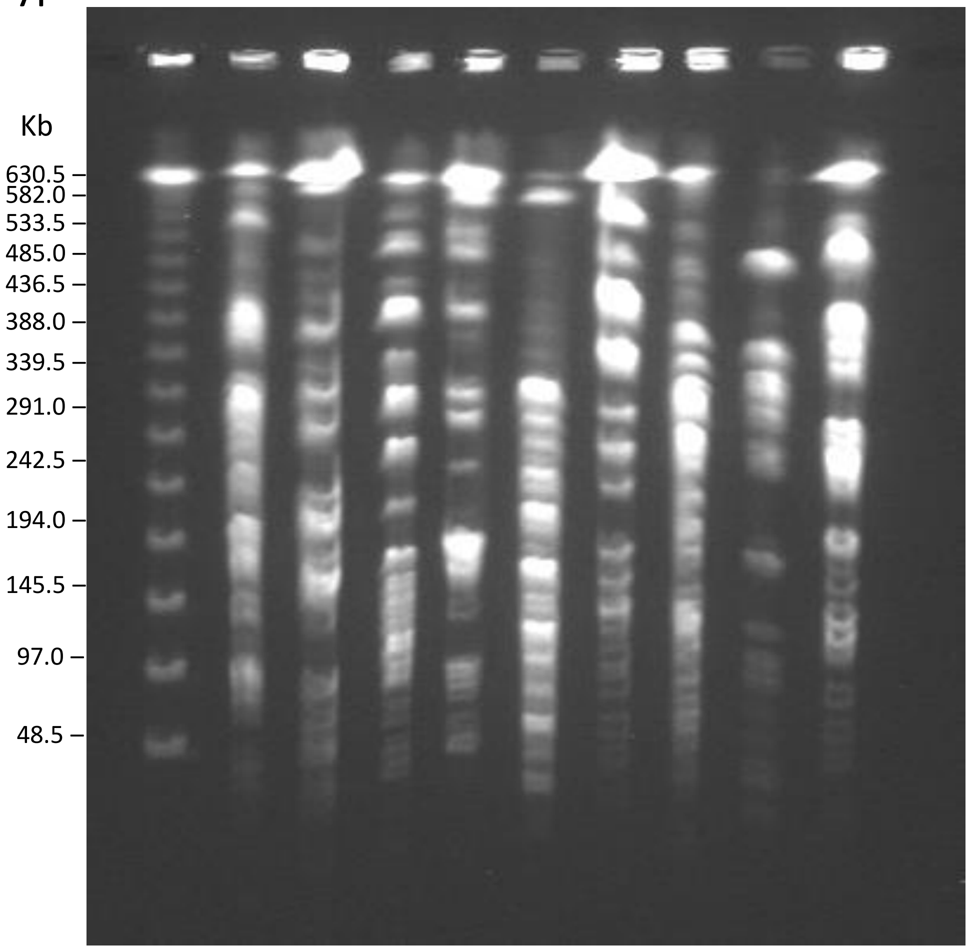

Isolate code E10 E11 E12 E13 E14 E15 E16 E17 E18

Molecular weight
PFGE lambda ladder

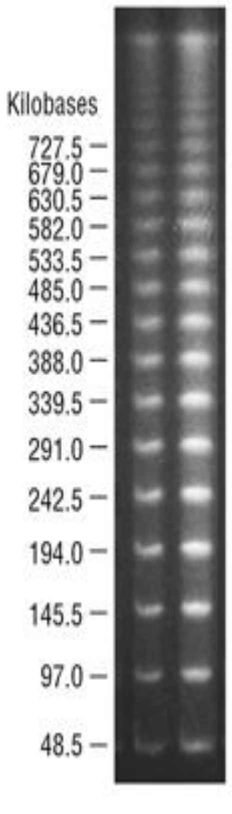

Gel 2 *Enterobacter*

Pulsotype **MW 2 4 5 MW**

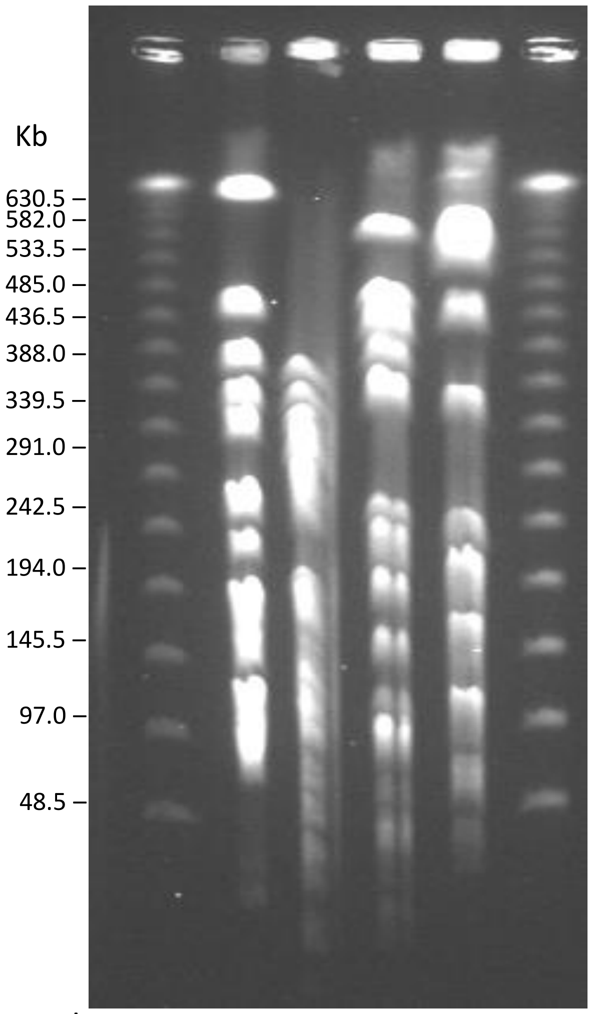

Isolate code E02 E03 E04 E05
