# Supplementary material for: Gram-negative ESKAPE bacteria bloodstream infections in patients during the COVID-19 pandemic
Source: PeerJ. 2023 Mar 29;11:e15007. doi: 10.7717/peerj.15007 (PMC10066687; doi:10.7717/peerj.15007)
Supplement: Supplemental Information 3 [file peerj-11-15007-s003.docx]

IDs GenBank

Strain: *Enterobacter cloacae*

Isolate number 11

Gene: beta-lactamase NDM-1

Acces number GenBank: OP738292

## Link for the third-party database: <https://www.ncbi.nlm.nih.gov/genbank/>

>7320_EntC11:1-193_Strain [organism=Enterobacter cloacae] strain C-11HGM2020, partial sequence

TCGGAATGGCTCATCACGATCATGCTGGCCTTGGGGAACGCCGCACCAAACGCGCGCGCTGACGCGGCGTAGTGCTCAGTGTCGGCATCACCGAGATTGCCGAGCGACTTGGCCTTGCTGTCCTTGATCAGGCAGCCACCAAAAGCGATGTCGGTGCCGTCGATCCCAACGGTGATATTGTCACTGGTGTGGCCGGGGCCGGGGTAAAATACCTTGAGCGGGCCAAAGTTGGGCGCGGTTGCTGGTTCGACCCAGCCATTGGCGGCGAAAGTCAGGCTGTGTTGCGCCGCAACCATCCCCTCTTGCGGGGCAAGCTGGTT**T**GACAACGCATTGGCATAAGTCGCAATCCCCGCCGCATGCAGCGCGTCCATACCGCCCATCTTGTCCTGATGCGCGTGAGTCACCACCGCCAGCGCGACCGGCAGGTTGATCTCCTGCTTGATCCAGTTGAGGATCTGGGCGGTCTGGTCATCGGTCCAGGCGGTATCGACCACCAGCACGCGGCCGCCATCCCTGACGATCAAACCGTTGGAAGCGACTGCCCCGAAACCCGGCATGTCGAGATAGGAAGTGTGCTGCCAGA

Strain: *Klebsiella pneumoniae*

Isolate number 24

Gene: beta-lactamase NDM-1

Acces number GenBank: OP738293

## Link for the third-party database: <https://www.ncbi.nlm.nih.gov/genbank/>

>7322_KpnC24:1-585 [organism=Klebsiella pneumoniae] strain C-24HGM2020, partial sequence

TCGGAATGGGGCTCATCACGATCATGCTGGCCTTGGGGAACGCCGCACCAAACGCGCGCGCTGACGCGGCGTAGTGCTCAGTGTCGGCATCACCGAGATTGCCGAGCGACTTGGCCTTGCTGTCCTTGATCAGGCAGCCACCAAAAGCGATGTCGGTGCCGTCGATCCCAACGGTGATATTGTCACTGGTGTGGCCGGGGCCGGGGTAAAAAACCTTGAGCGGGCCAAAGTTGGGCGCGGTTGCTGGTTCGACCCAGCCATTGGCGGCGAAAGTCAGGCTGTGTTGCGCCGCAACCATCCCCTCTTGCGGGGCAAGCTGGTTTGACAACGCATTGGCATAAGTCGCAATCCCCGCCGCATGCAGCGCGTCCATACCGCCCATCTTGTCCTGATGCGCGTGAGTCACCACCGCCAGCGCGACCGGCAGGTTGATCTCCTGCTTGATCCAGTTGAGGATCTGGGCGGTCTGGTCATCGGTCCAGGCGGTATCGACCACCAGCACGCGGCCGCCATCCCTGACGATCAAACCGTTGGAAGCGACTGCCCCGAAACCCGGCATGTCGAGATAGGAAGTGTGCTGCCAGA

Strain: *Pseudomonas aeruginosa*

Isolate number 05

Gene: beta-lactamase VIM-2

Acces number GenBank: OP738291

## Link for the third-party database: <https://www.ncbi.nlm.nih.gov/genbank/>

>7325_PsaC05:1-346_Strain [organism=Pseudomonas aeruginosa] strain C-05HGM2020, partial sequence

GGTGTTTGGTCGCATATCGCAACGCAGTCGTTTGATGGCGCAGTCTACCCGTCCAATGGTCTCATTGTCCGTGATGGTGATGAGTTGCTTTTGATTGATACAGCGTGGGGTGCGAAAAACACAGCGGCACTTCTCGCGGAGATTGAGAAGCAAATTGGACTTCCTGTAACGCGTGCAGTCTCCACGCACTTTCATGACGACCGCGTCGGCGGCGTTGATGTCCTTCGGGCGGCTGGGGTGGCAACGTACGCATCACCGTCGACACGCCGGCTAGCCGAGGTAGAGGGGAACGAGATTCCCACGCACTCTCTAGAAGGACTCTCATCGAGCGGGACGCAGTGCGCTC

Strain: *Klebsiella pneumoniae*

Isolate number 24

Gene: beta-lactamase TEM-1

Acces number GenBank: OP738294

## Link for the third-party database: <https://www.ncbi.nlm.nih.gov/genbank/>

>7327_KpnC24:1-637_Strain [organism=Klebsiella pneumoniae] strain C-24HGM2020, partial sequence

AACGTTTTCCAATGATGAGCACTTTTAAAGTTCTGCTATGTGGTGCGGTATTATCCCGTGTTGACGCCGGGCAAGAGCAACTCGGTCGCCGCATACACTATTCTCAGAATGACTTGGTTGAGTACTCACCAGTCACAGAAAAGCATCTTACGGATGGCATGACAGTAAGAGAATTATGCAGTGCTGCCATAACCATGAGTGATAACACTGCTGCCAACTTACTTCTGACAACGATCGGAGGACCGAAGGAGCTAACCGCTTTTTTGCACAACATGGGGGATCATGTAACTCGCCTTGATCGTTGGGAACCGGAGCTGAATGAAGCCATACCAAACGACGAGCGTGACACCACGATGCCTGCAGCAATGGCAACAACGTTGCGCAAACTATTAACTGGCGAACTACTTACTCTAGCTTCCCGGCAACAATTAATAGACTGGATGGAGGCGGATAAAGTTGCAGGACCACTTCTGCGCTCGGCCCTTCCGGCTGGCTGGTTTATTGCTGATAAATCTGGAGCCGGTGAGCGTGGGTCTCGCGGTATCATTGCAGCACTGGGGCCAGATGGTAAGCCCTCCCGTATCGTAGTTATCTACACGACGGGGAGTCAGGCAACTATGGATGAACGAAATAGACA

Strain: *Klebsiella pneumoniae*

Isolate number 09

Gene: beta-lactamase TEM-1

Acces number GenBank: OP738295

## Link for the third-party database: <https://www.ncbi.nlm.nih.gov/genbank/>

>7329_KpnC09:1-778_Strain [organism=Klebsiella pneumoniae] strain C-09HGM2020, partial sequence

TGTCTATTTCGTTCATCCATAGTTGCCTGACTCCCCGTCGTGTAGATAACTACGATACGGGAGGGCTTACCATCTGGCCCCAGTGCTGCAATGATACCGCGAGACCCACGCTCACCGGCTCCAGATTTATCAGCAATAAACCAGCCAGCCGGAAGGGCCGAGCGCAGAAGTGGTCCTGCAACTTTATCCGCCTCCATCCAGTCTATTAATTGTTGCCGGGAAGCTAGAGTAAGTAGTTCGCCAGTTAATAGTTTGCGCAACGTTGTTGCCATTGCTGCAGGCATCGTGGTGTCACGCTCGTCGTTTGGTATGGCTTCATTCAGCTCCGGTTCCCAACGATCAAGGCGAGTTACATGATCCCCCATGTTGTGCAAAAAAGCGGTTAGCTCCTTCGGTCCTCCGATCGTTGTCAGAAGTAAGTTGGCAGCAGTGTTATCACTCATGGTTATGGCAGCACTGCATAATTCTCTTACTGTCATGCCATCCGTAAGATGCTTTTCTGTGACTGGTGAGTACTCAACCAAGTCATTCTGAGAATAGTGTATGCGGCGACCGAGTTGCTCTTGCCCGGCGTCAACACGGGATAATACCGCACCACATAGCAGAACTTTAAAAGTGCTCATCATTGGAAAACGTTCTTCGGGGCGAAAACTCTCAAGGATCTTACCGCTGTTGAGATCCAGTTCGATGTAACCCACTCGTGCACCCAACTGATCTTCAGCATCTTTTACTTTCACCAGCGTTTCTGGGTGAGCAAAAACAGGAAGGCAAAATGCCG

Strain: *Klebsiella pneumoniae*

Isolate number 24

Gene: beta-lactamase CTXM-15

Acces number GenBank: OP738296

## Link for the third-party database: <https://www.ncbi.nlm.nih.gov/genbank/>

## >7331_Kpn_C24

CAGTCGGCTATCCCCCACAACCCAGGAAGCAGGCAGTCCAGCCTGAATGCTCGCTGCACCGGTGGTATTGCCTTTCATCCATGTCACCAGCTGCGCCCGTTGGCTGTCGCCCAATGCTTTACCCAGCGTCAGATTCCGCAGAGTTTGCGCCATTGCCCGAGGTGAAGTGGTATCACGCGGATCGCCCGGAATGGCGGTGTTTAACGTCGGCTCGGTACGGTCGAGACGGAACGTTTCGTCTCCCAGCTGTCGGGCGAACGCGGTGACGCTAGCCGGGCCGCCAACGTGAGCAATCAGCTTATTCATCGCCACGTTATCGCTGTACTGTAGCGCGGCCGCGCTAAGCTCAGCCAGTGACATCGTCCCATTGACGTGCTTTTCCGCAATCGGATTATAGTTAACAAGGTCAGATTTTTTGATCTCAACTCGCTGATTTAACAGATTCGGTTCGCTTTCACTTTTCTTCAGCACCGCGGCCGCGGCCATCACTTTACTGGTGCTGCACATCGCAAAGCGAGG

Strain: *Klebsiella pneumoniae*

Isolate number 12

Gene: beta-lactamase CTXM-15

Acces number GenBank: OP738297

## Link for the third-party database: <https://www.ncbi.nlm.nih.gov/genbank/>

>7333_Kpn_C12

AGTCAGCCTTATCCCCACAACCCAGGAAGCAGGCAGTCCAGCCTGAATGCTCGCTGCACCGGTGGTATTGCCTTTCATCCATGTCACCAGCTGCGCCCGTTGGCTGTCGCCCAATGCTTTACCCAGCGTCAGATTCCGCAGAGTTTGCGCCATTGCCCGAGGTGAAGTGGTATCACGCGGATCGCCCGGAATGGCGGTGTTTAACGTCGGCTCGGTACGGTCGAGACGGAACGTTTCGTCTCCCAGCTGTCGGGCGAACGCGGTGACGCTAGCCGGGCCGCCAACGTGAGCAATCAGCTTATTCATCGCCACGTTATCGCTGTACTGTAGCGCGGCCGCGCTAAGCTCAGCCAGTGACATCGTCCCATTGACGTGCTTTTCCGCAATCGGATTATAGTTAACAAGGTCAGATTTTTTGATCTCAACTCGCTGATTTAACAGATTCGGTTCGCTTTCACTTTTCTTCAGCACCGCGGCCGCGGCCATCACTTTACTGGTGCTGCACATCGCAAAGCG

Strain: *Acinetobacter baumannii*

Isolate number 07

Gene: beta-lactamase TEM-1

Acces number GenBank: OP745941

## Link for the third-party database: <https://www.ncbi.nlm.nih.gov/genbank/>

>10079_AbaC07:1-781_Strain [organism=Acinetobacter baumannii] strain Aba_C-07HGM2020, partial sequence

TCTGTCTATTTCGTTCATCCATAGTTGCCTGACTCCCCGTCGTGTAGATAACTACGATACGGGAGGGCTTACCATCTGGCCCCAGTGCTGCAATGATACCGCGAGATCCACGCTCACCGGCTCCAGATTTATCAGCAATAAACCAGCCAGCCGGAAGGGCCGAGCGCAGAAGTGGTCCTGCAACTTTATCCGCCTCCATCCAGTCTATTAATTGTTGCCGGGAAGCTAGAGTAAGTAGTTCGCCAGTTAATAGTTTGCGCAACGTTGTTGCCATTGCTGCAGGCATCGTGGTGTCACGCTCGTCGTTTGGTATGGCTTCATTCAGCTCCGGTTCCCAACGATCAAGGCGGGTTACATGATCCCCCATGTTGTGCAAAAAAGCGGTTAGCTCCTTCGGTCCTCCGATCGTTGTCAGAAGTAAGTTGGCCGCAGTGTTATCACTCATGGTTATGGCAGCACTGCATAATTCTCTTACTGTCATGCCATCCGTAAGATGCTTTTCTGTGACTGGTGAGTACTCAACCAAGTCATTCTGAGAATAGTGTATGCGGCGACCGAGTTGCTCTTGCCCGGCGTCAACACGGGATAATACCGCACCACATAGCAGAACTTTAAAAGTGCTCATCATTGGAAAACGTTCTTCGGGGCGAAAACTCTCAAGGATCTTACCGCTGTTGAGATCCAGCTCGATGTAACCCACTCGTGCACCCAACTGATCTTCAGCATCTTTTACTTTCACCAGCGTTTCTGGGTGAGCAAAAACAGGAAGGCAAAATGCCGC

Strain: *Acinetobacter baumannii*

Isolate number 32

Gene: beta-lactamase TEM-1

Acces number GenBank: OP745942

## Link for the third-party database: <https://www.ncbi.nlm.nih.gov/genbank/>

>10081_AbaC32:1-781_Strain [organism=Acinetobacter baumannii] strain Aba_C-32HGM2020, partial sequence

TTTTTTGCGGCATTTTGCCTTCCTGTTTTTGCTCACCCAGAAACGCTGGTGAAAGTAAAAGATGCTGAAGATCAGTTGGGTGCACGAGTGGGTTACATCGAGCTGGATCTCAACAGCGGTAAGATCCTTGAGAGTTTTCGCCCCGAAGAACGTTTTCCAATGATGAGCACTTTTAAAGTTCTGCTATGTGGTGCGGTATTATCCCGTGTTGACGCCGGGCAAGAGCAACTCGGTCGCCGCATACACTATTCTCAGAATGACTTGGTTGAGTACTCACCAGTCACAGAAAAGCATCTTACGGATGGCATGACAGTAAGAGAATTATGCAGTGCTGCCATAACCATGAGTGATAACACTGCGGCCAACTTACTTCTGACAACGATCGGAGGACCGAAGGAGCTAACCGCTTTTTTGCACAACATGGGGGATCATGTAACCCGCCTTGATCGTTGGGAACCGGAGCTGAATGAAGCCATACCAAACGACGAGCGTGACACCACGATGCCTGCAGCAATGGCAACAACGTTGCGCAAACTATTAACTGGCGAACTACTTACTCTAGCTTCCCGGCAACAATTAATAGACTGGATGGAGGCGGATAAAGTTGCAGGACCACTTCTGCGCTCGGCCCTTCCGGCTGGCTGGTTTATTGCTGATAAATCTGGAGCCGGTGAGCGTGGATCTCGCGGTATCATTGCAGCACTGGGGCCAGATGGTAAGCCCTCCCGTATCGTAGTTATCTACACGACGGGGAGTCAGGCAACTATGGATGAACGAAATAGACA

Strain: *Acinetobacter baumannii*

Isolate number 34

Gene: beta-lactamase TEM-1

Acces number GenBank: OP745943

## Link for the third-party database: <https://www.ncbi.nlm.nih.gov/genbank/>

>10082_AbaC34:1-754_Strain [organism=Acinetobacter baumannii] strain Aba_C-34HGM2020, partial sequence

GCCTTCCTGTTTTTGCTCACCCAGAAACGCTGGTGAAAGTAAAAGATGCTGAAGATCAGTTGGGTGCACGAGTGGGTTACATCGAACTGGATCTCAACAGCGGTAAGATCCTTGAGAGTTTTCGCCCCGAAGAACGTTTTCCAATGATGAGCACTTTTAAAGTTCTGCTATGTGGTGCGGTATTATCCCGTGTTGACGCCGGGCAAGAGCAACTCGGTCGCCGCATACACTATTCTCAGAATGACTTGGTTGAGTACTCACCAGTCACAGAAAAGCATCTTACGGATGGCATGACAGTAAGAGAATTATGCAGTGCTGCCATAACCATGAGTGATAACACTGCTGCCAACTTACTTCTGACAACGATCGGAGGACCGAAGGAGCTAACCGCTTTTTTGCACAACATGGGGGATCATGTAACTCGCCTTGATCGTTGGGAACCGGAGCTGAATGAAGCCATACCAAACGACGAGCGTGACACCACGATGCCTGCAGCAATGGCAACAACGTTGCGCAAACTATTAACTGGCGAACTACTTACTCTAGCTTCCCGGCAACAATTAATAGACTGGATGGAGGCGGATAAAGTTGCAGGACCACTTCTGCGCTCGGCCCTTCCGGCTGGCTGGTTTATTGCTGATAAATCTGGAGCCGGTGAGCGTGGGTCTCGCGGTATCATTGCAGCACTGGGGCCAGATGGTAAGCCCTCCCGTATCGTAGTTATCTACACGACGGGGAGTCAGGCAACTATGGA

Strain: *Acinetobacter baumannii*

Isolate number 34

Gene: beta-lactamase OXA-24

Acces number GenBank: OP745944

## Link for the third-party database: <https://www.ncbi.nlm.nih.gov/genbank/>

>10085_AbaC34:1-754_Strain [organism=Acinetobacter baumannii] strain Aba_C-34HGM2020, partial sequence

TTTCTAAGTTGAGCGAAAAGGGGATTTTTTTTCC**A**TTAGCTTGCTCCACCCAACCAGTCAACCAACCTACCTGTGGAGTAACATCCATTCCCCATCCACTTTTTGCATAAATCTTACTACCATTTACTTCTTTAATTAGAAGCATTTTTTTAACTTCTTCTTGAGTTTCTAATTTAAAAGGTAATCGGTTATGTGCAAGGTCATCGGCAAAATTAACTTCTTGTACTGGTGTAATTTTAAGGGGGCCAACTAACCAAAAATTATCGACCTGTGTTCCAATATTTGTATTTCCAAAATTAACCCGCTTTACTTCTTTCTGCATTAGCTCTAGGCCAGTCCGTCTTGCAAGCTCTTGATATACTGGAACTGCTGACAATGCCATTGCCTCACCTAAAGTCATATCTTTCTCCCACATAGGATAAGTTCTTTTTTTACCATCCCATTT**G**AAAATCTCATTTGTTGTTGCTTTATGATTTTCTAGCCCGATTAAAGCATTTAGCATCTTAAATGTTGATGCAGGGACATATTCTTTATTTGCTCGTGCAAGAGCATTACCATAGGTGCTAAGATTTTTACCCTCTTTAATAATAATTACACCCTGTGTTTGAGCTTCATCAAAATAGCTTTTAATAGCTTTTTCATGTTGCTGAGAAGAAATAT**G**AAAATTATCTTCAGATTTAGTTTTAATA

Strain: *Acinetobacter baumannii*

Isolate number 10

Gene: beta-lactamase OXA-24

Acces number GenBank: OP745945

## Link for the third-party database: <https://www.ncbi.nlm.nih.gov/genbank/>

>10087_AbaC10:1-714_Strain [organism=Acinetobacter baumannii] strain Aba_C-10HGM2020, partial sequence

TTCTAAGTTGAGCGAAAAGGGGATTTTTTTTCC**A**TTAGCTTGCTCCACCCAACCAGTCAACCAACCTACCTGTGGAGTAACATCCATTCCCCATCCACTTTTTGCATAAATCTTACTACCATTTACTTCTTTAATTAGAAGCATTTTTTTAACTTCTTCTTGAGTTTCTAATTTAAAAGGTAATCGGTTATGTGCAAGGTCATCGGCAAAATTAACTTCTTGTACTGGTGTAATTTTAAGGGGGCCAACTAACCAAAAATTATCGACCTGTGTTCCAATATTTGTATTTCCAAAATTAACCCGCTTTACTTCTTTCTGCATTAGCTCTAGGCCAGTCCGTCTTGCAAGCTCTTGATATACTGGAACTGCTGACAATGCCATTGCCTCACCTAAAGTCATATCTTTCTCCCACATAGGATAAGTTCTTTTTTTACCATCCCATTTGAAAATCTCATTTGTTGTTGCTTTATGATTTTCTAGCCCGATTAAAGCATTTAGCATCTTAAATGTTGATGCAGGGACATATTCTTTATTTGCTCGTGCAAGAGCATTACCATAGGTGCTAAGATTTTTACCCTCTTTAATAATAATTACACCCTGTGTTTGAGCTTCATCAAAATAGCTTTTAATAGCTTTTTCATGTTGCTGAGAAGAAATAT**G**AAAATTATCTTCAGATTTAGTTTTAATAGATGAACATGCACTGAGAGAAACTAG

Strain: *Acinetobacter baumannii*

Isolate number 07

Gene: beta-lactamase OXA-398

Acces number GenBank: OP745946

## Link for the third-party database: <https://www.ncbi.nlm.nih.gov/genbank/>

>10089_AbaC07:1-712_Strain [organism=Acinetobacter baumannii] strain Aba_C-07HGM2020, partial sequence

GCGACAATTTTTCCATCTGGCTGCTCAACCCAGCCGGTCAACCAGCCCACTTGTTGTTTTATATCCATTGCCCAACCAGTCTTTCCAAAAATTTTGTAGCCATTACTCTCTTCTAAAAGAAGCATATTTTTTACATTAGCCTGCACTTTTTCACTAAATGGAAGCTGTGTATGTGCTAATTGGGAAACAAACTCTACCTCTTGAATAGGCGTAACCTTTAATGGTCCTACCAACCAGAAATTATCAACCTGCTGTCCAATTTCAGCATTACCGAAACCAATACGTTTTACTTCTTTTTGCATGAGATCAAGACCGATACGTCGCGCAAGTTCCTGATAGACTGGGACTGCAGAAAGCTTCATGGCTTCTCCTAGTGTCATGTCTTTTTCCCAAGCGGTAAATGACCTTTTCTCGCCCTTCCATTTAAATATTTCATTAATATCCGTTTTCTGGTTCTCCAATCCGATCAGGGCATTCAACATTTTAAATGTAGAGGCTGGCACATATTCTGTATTTGCGCGGCTTAGAGCATTACCATATAGATTAATTTTTTTATCTGTTTGAATAACCAGCACACCTGAGGTGTTTTTTTCATCAAAGTATTGATGAATCACCTGATTATGTCCTTGAACAATCTGACTCGGGGTTTCATTTATTAAATTATGCTGAACCGTACAACCAGAAAGAAAAAGAGAAGCAACCACATAGCAAG

Strain: *Acinetobacter baumannii*

Isolate number 32

Gene: beta-lactamase OXA-398

Acces number GenBank: OP745947

## Link for the third-party database: <https://www.ncbi.nlm.nih.gov/genbank/>

>10091_AbaC32:1-725_Strain [organism=Acinetobacter baumannii] strain Aba_C-32HGM2020, partial sequence

ATGCAAAAGCGACAATTTTTCCATCTGGCTGCTCAACCCAGCCGGTCAACCAGCCCACTTGTTGTTTTATATCCATTGCCCAACCAGTCTTTCCAAAAATTTTGTAGCCATTACTCTCTTCTAAAAGAAGCATATTTTTTACATTAGCCTGCACTTTTTCACTAAATGGAAGCTGTGTATGTGCTAATTGGGAAACAAACTCTACCTCTTGAATAGGCGTAACCTTTAATGGTCCTACCAACCAGAAATTATCAACCTGCTGTCCAATTTCAGCATTACCGAAACCAATACGTTTTACTTCTTTTTGCATGAGATCAAGACCGATACGTCGCGCAAGTTCCTGATAGACTGGGACTGCAGAAAGCTTCATGGCTTCTCCTAGTGTCATGTCTTTTTCCCAAGCGGTAAATGACCTTTTCTCGCCCTTCCATTTAAATATTTCATTAATATCCGTTTTCTGGTTCTCCAATCCGATCAGGGCATTCAACATTTTAAATGTAGAGGCTGGCACATATTCTGTATTTGCGCGGCTTAGAGCATTACCATATAGATTAATTTTTTTATCTGTTTGAATAACCAGCACACCTGAGGTGTTTTTTTCATCAAAGTATTGATGAATCACCTGATTATGTCCTTGAACAATCTGACTCGGGGTTTCATTTATTAAATTATGCTGAACCGTACAACCAGAAAGAAAAAGAGAAGCAACCACATAGCAAGTAAAA
